# Subtype-Selective Small Molecule Inhibitors Reveal a Fundamental Role for Nav1.7 in Nociceptor Electrogenesis, Axonal Conduction and Presynaptic Release

**DOI:** 10.1371/journal.pone.0152405

**Published:** 2016-04-06

**Authors:** Aristos J. Alexandrou, Adam R. Brown, Mark L. Chapman, Mark Estacion, Jamie Turner, Malgorzata A. Mis, Anna Wilbrey, Elizabeth C. Payne, Alex Gutteridge, Peter J. Cox, Rachel Doyle, David Printzenhoff, Zhixin Lin, Brian E. Marron, Christopher West, Nigel A. Swain, R. Ian Storer, Paul A. Stupple, Neil A. Castle, James A. Hounshell, Mirko Rivara, Andrew Randall, Sulayman D. Dib-Hajj, Douglas Krafte, Stephen G. Waxman, Manoj K. Patel, Richard P. Butt, Edward B. Stevens

**Affiliations:** 1 Pfizer Neusentis, The Portway Buiding, Granta Park, Great Abington, Cambridge, CB21 6GS, United Kingdom; 2 Pfizer Neusentis, 4222 Emperor Boulevard, Durham, North Carolina, 27703, United States of America; 3 Dept. Anesthesiology, University of Virginia Health System, Charlottesville, Virginia, 22911, United States of America; 4 Worldwide Medicinal Chemistry, Pfizer Neusentis, The Portway Building, Granta Park, Great Abington, Cambridge, CB21 6GS, United Kingdom; 5 Pfizer Global R&D, Ramsgate Road, Sandwich, Kent, CT13 9NJ, United Kingdom; 6 Center for Neuroscience & Regeneration Research, Yale Medical School and Veterans Affairs Hospital, West Haven, CT, 06516, United States of America; 7 Medical School, Hatherly Building, University of Exeter, Prince of Wales Road, Exeter, EX4 4PS, United Kingdom; University of Texas at Dallas, UNITED STATES

## Abstract

Human genetic studies show that the voltage gated sodium channel 1.7 (Na_v_1.7) is a key molecular determinant of pain sensation. However, defining the Na_v_1.7 contribution to nociceptive signalling has been hampered by a lack of selective inhibitors. Here we report two potent and selective arylsulfonamide Na_v_1.7 inhibitors; PF-05198007 and PF-05089771, which we have used to directly interrogate Na_v_1.7’s role in nociceptor physiology. We report that Na_v_1.7 is the predominant functional TTX-sensitive Na_v_ in mouse and human nociceptors and contributes to the initiation and the upstroke phase of the nociceptor action potential. Moreover, we confirm a role for Na_v_1.7 in influencing synaptic transmission in the dorsal horn of the spinal cord as well as peripheral neuropeptide release in the skin. These findings demonstrate multiple contributions of Na_v_1.7 to nociceptor signalling and shed new light on the relative functional contribution of this channel to peripheral and central noxious signal transmission.

## Introduction

Numerous genetic studies implicate Na_v_1.7 in the pathogenesis of distinct pain states (for reviews see [1] [2]). In particular, loss-of-function mutations in SCN9A (the gene encoding Na_v_1.7) have been identified in patients with congenital insensitivity to pain (CIP; [3]), whereas gain-of-function mutations in SCN9A lead to chronic pain syndromes such as paroxysmal extreme pain disorder (PEPD, [4]) and inherited erythromelalgia (IEM) [5] [6] [7] [8]. Moreover, expression of Na_v_1.7 in DRG neurons extends from the peripheral terminals in the skin to the central terminals in the dorsal horn [9]. These studies present a clear link between Na_v_1.7 function and pain sensation and raise the possibility that selective Na_v_1.7 inhibitors might hold therapeutic potential as novel analgesics.

Despite the strong evidence implicating Na_v_1.7 in human pain genetic studies, a detailed investigation of the role of Na_v_1.7 in nociception remains an important area of investigation. Na_v_ channels are essential for action potential initiation and upstroke in excitable cells. Out of a repertoire of nine Na_v_s (Na_v_1.1–1.9), five are expressed in varying levels in adult rodent somatosensory DRG neurons: Na_v_1.1, Na_v_1.6, Na_v_1.7, Na_v_1.8 and Na_v_1.9 [10] [11] [12] [13]. Given the participation of multiple Na_v_s in pain signalling, progress in delineating the individual roles of specific Na_v_ isoforms in DRG neurons would be accelerated if subtype-selective inhibitors were available.

In this study we characterize two novel arylsulfonamides: a clinical compound, PF-05089771 (for a list of relevant clinical trials see [14]) and a structurally related preclinical tool compound, PF-05198007. Both demonstrate high potency and a high degree of Na_v_ subtype selectivity, properties which are gained through a drug/channel interaction that is distinct from that of the classical non-selective pore-blocking drugs such as local anaesthetics. We examined the effects of selective Na_v_1.7 block in both *in vitro* and *in vivo* preparations with the principal aim of exploring how Na_v_1.7 influences nociceptor function. Our findings establish a mechanistic basis for Na_v_1.7 contribution to action potential electrogenesis in small diameter DRG neurons and describe a functional role for Na_v_1.7 in controlling both neuropeptide release in the peripheral compartment and synaptic transmission in the dorsal horn of the spinal cord.

## Materials & Methods

### Cell culture

Human embryonic kidney (HEK) 293 cells stably expressing human and mouse Na_v_ subtypes were commercially obtained (Millipore). Cells were maintained using minimum essential medium (MEM) with Earle’s salts supplemented by 10% foetal calf serum, 2 mM L-glutamine, 1 mM sodium pyruvate, 1x non-essential amino acids and 0.4 mg/ml geneticin (G-418) and kept at 37°C in a humidified atmosphere of 5% CO_2_. For manual patch clamp experiments, cells were plated onto glass coverslips and used within 48 hours.

### Ethical Approval

Mice were killed by cervical dislocation in accordance with Schedule 1 of the UK Government Animals (Scientific Procedures) Act, 1986, following approval by the Animal Welfare and Ethical Review Body, or euthanized by isoflurane in accordance with the National Institutes of Health guide for the Care and Use of Animals following approval by the University of Virginia Institute of Animal Care and Use Committee.

### Mouse DRG preparation

Dorsal root ganglia (DRG) were isolated and dissociated according to a previously published method [15]. Briefly, DRGs were obtained from all spinal locations and dissociated neurons plated on glass coverslips pre-coated with poly-D-lysine/laminin (BD Biosciences) and left to adhere for 1.5–2 hrs before flooding. Growth media consisted of Lebovitz L-15 Glutamax (Life Technologies) supplemented with 10% FCS, 24 mM NaHCO_3_ and 38 mM glucose.

### Human DRG preparation

Human DRGs (hDRGs) were surgically resected from US organ donors with full legal consent. The hDRG culturing process has been previously described in detail [16]. Briefly, DRG neurons were enzymatically dissociated and maintained in culture for up to 9 days prior to recording. All hDRG tissue culture and experiments on hDRG neurons were conducted at Anabios Corporation (San Diego, CA, USA).

### Molecular Biology/ RNASeq

Approximately 300 small diameter neurons per sample (n = 4 animals, 2 samples per animal) were picked off coverslips in PBS-/- (phosphate buffered saline without calcium or magnesium) (Invitrogen) using a patch pipette. RNA was extracted using the Qiagen RNeasy micro kit as per the manufacturer’s protocol, including on-column DNase digestion. These control samples were then converted to cDNA and amplified for 18 cycles using the SMARTer v3 kit (Clontech). Libraries were quantified using the Nextera XT kit (Illumina), following a modified protocol (see Fluidigm C1 Single-Cell Auto Prep System manual). This protocol uses lower input amounts than the standard Illumina method; 1.25 μl cDNA at 0.1 ng/μl per tagmentation reaction (5 μl total reaction volume). Negative controls were also prepared from each animal by collecting bath solution; these produced no product at the end of amplification or library preparation and hence were not sequenced. Libraries were quantitated individually using the Qubit High Sensitivity DNA assay (Thermo Fisher Scientific) and library quantification kits (KAPA Biosystems) and pooled in equal amounts for single-end sequencing on an Illumina Nextseq 500. Reads were aligned using STAR (spliced transcripts alignment and reconstruction tool) and assigned to genomic features using Counts and Ensembl gene annotations. Gene read counts were converted to fragments per kilobase per million (FPKM).

### Electrophysiology

Electrophysiological recordings were obtained from stably transfected HEK 293 cells, acutely dissociated mouse DRG neurons, cultured human DRG neurons or mouse superficial dorsal horn neurons using either an Axopatch 200B or Axopatch 700B amplifier (Molecular Devices), or EPC-10 USB amplifier (HEKA). Small diameter mouse DRG neurons were identified as those with a whole cell capacitance between 10–25 pF [17]. For human DRG neurons, patch clamp recordings were obtained from small-to-medium diameter neurons (<60 μm), the majority of which have previously been classified as nociceptors [16].

Patch pipettes were pulled from thick-walled borosilicate glass (Science Products) and had open tip resitances of 1.5–5 MΩ when filled with intracellular solution (ICS; see below for composition). Data acquisition was performed using either pClamp v10 (Molecular Devices) or PatchMaster (HEKA) software.

#### Voltage clamp

HEK cells or mouse DRG neurons were continuously superfused with extracellular solution (ECS) containing (in mM): 30 NaCl, 110 Choline Cl, 3 KCl, 0.8 MgCl_2_, 1.8 CaCl_2_, 0.05 CdCl_2_, 10 Glucose, 10 HEPES, 5 Sucrose (300–310 mOsm, titrated to pH 7.4 with TEA-OH). The patch pipette (intracellular) solution (ICS) contained (in mM): 5 NaCl, 135 CsF, 10 CsCl, 2 MgATP, 10 HEPES, 5 EGTA (290–300 mOsm, titrated to pH 7.2 with KOH). For human DRG recordings the following solutions were used (ECS in mM):150 NaCl, 4 BaCl, 2 CaCl_2_, 1 MgCl_2_, 0.1 CdCl_2_, 10 Glucose, 10 HEPES, (300–310 mOsm titrated to pH 7.3 with Na-OH). ICS in mM: 140 CsF, 10 NaCl, 1 EGTA, 1 MgCl_2,_ 10 HEPES, 10 glucose, (290–300 mOsm, titrated to pH 7.3 with Cs-OH). Series resistance compensation was routinely applied to at least 75%. Before acquisition, 20 ms pulses to 0 mV were repeatedly applied (0.05 Hz) from Vm = -120 mV until stable current responses were obtained. All experiments were carried out at room temperature (21–24°C). IC_50_ values were generated in HEK 293 cell lines by voltage clamping at -120 mV before stepping to the V_0.5_ of inactivation for 5 seconds in order to accumulate compound binding. This was followed by a 100 ms return to -120 mV preceding a 20 ms test step to 0 mV. Cells with large TTX-S currents (>5 nA mouse, >8 nA human) and cells with series resistance values greater than 15 MΩ, or variable series resistance were omitted from analysis.

#### Current clamp

ECS contained (in mM) 135 NaCl, 4.7 KCl, 1 CaCl2, 1 MgCl2, 10 HEPES and 10 glucose (300–310 mOsm, pH 7.4 with NaOH). ICS contained (in mM) 140 KCl, 0.5 EGTA, 5 HEPES, and 3 Mg-ATP, (pH 7.3 with KOH, 300–305 mOsm). Single action potentials were evoked from V_m_ -70 mV by a 20 ms suprathreshold current step (0.1 Hz).

#### Action potential voltage clamp

Recordings were performed as described in Blair & Bean (2002). Briefly, the internal solution contained (in mM): 140 K-Mes, 13.5 NaCl, 1.6 MgCl_2_, 0.09 EGTA, 9 HEPES, 0.9 glucose, 14 Tris-creatine PO_4_, 4 Mg-ATP, and 0.3 Tris-GTP (285–300 mOsm, pH 7.2 with KOH). The ECS was identical to that used for current clamp recordings (see above). Action potentials were recorded in current clamp mode following a 20 ms current injection from a V_m_ of -70 mV. The same cell was then examined under voltage-clamp (-70 mV) whereby the recorded action potential was used as the voltage command. Series resistance was compensated for by up to 80%.

### PatchXpress automated electrophysiological recordings

Extracellular solution contained (in mM): 40–138 NaCl, 0–98 choline chloride, 2 CaCl_2_, 5.4 KCl, 1 MgCl_2_, 10 glucose, and 10 HEPES, (295–310 mOsm, pH 7.4 with NaOH). Internal solution contained (in mM): 135 CsF, 5 NaCl, 2 MgCl2, 10 EGTA, 10 HEPES (285–295 mOsm, pH 7.3 with NaOH). HEK 293 cells constitutively expressing human sodium channels were grown as above to 50–80% confluency and harvested by trypsinization. Trypsinized cells were washed and resuspended in extracellular buffer at a concentration of 10^6^ cells/ml. The onboard liquid handling facility of the PatchXpress was used for dispensing cells and application of test compounds, where washout periods were limited to a maximal duration of 5–10 min. The effect of PF-05089771 was evaluated with voltage protocols identical to those used for conventional patch clamp. A detailed rationale and description of the development of the PatchXpress protocol has been previously described [18]. Previous characterization of the PatchXpress platform demonstrated the ability to generate Na_v_ channel pharmacology comparable to conventional patch clamp data[18].

### Spinal cord slice preparation and electrophysiology

Male adult CD1 mice were terminally euthanized and the spinal column was removed and submerged in chilled (4°C) artificial cerebrospinal fluid solution (aCSF) comprised (in mM): 125 NaCl, 25 NaHCO_3_, 10 glucose, 2.5 KCl, 1.25 NaH_2_PO_4_, 2 CaCl_2_, 1 MgCl_2_, 0.5 L-ascorbic acid and 2 pyruvate (300–310 mOsm, bubbled with a 95%, 5% O_2_/CO_2_ gas mixture). Following lumbosacral laminectomy, the dura mater and pia-arachnoid membrane were carefully removed leaving intact dorsal roots. Para-saggital slices (400 μm) were prepared using a Vibratome 1000 Plus sectioning system (Vibratome) and stored in warm oxygenated (37°C) aCSF for 35 mins after which slices were kept at room temperature until required. Slices were transferred to a recording chamber and perfused at 2 mL/min with aCSF heated to 32°C. For experiments in which test compound was applied to the dorsal roots only, a recording chamber was used that consisted of two separate baths, each with their own perfusion inlets and outlets. The separate chambers were connected via tunnels that allowed the dorsal root to pass across and was then sealed using silicone grease. Both chambers were independently perfused at 2 mL/min with aCSF heated to 32°C. Whole-cell voltage clamp recordings were obtained from visually identified substantia gelatinosa neurons present in lamina II. ICS containied (in mM): 120 K gluconate, 10 NaCl, 2 MgCl_2_, 0.5 K_2_EGTA, 10 HEPES, 4 Na_2_ATP, 0.3 NaGTP, 20 biocytin (285–300 mOsm, pH adjusted to 7.2 with KOH).

Synaptic responses were evoked using a glass suction electrode to deliver a 400 μs stimulus of varying current amplitude (1 to 3.2 mA) applied every 15 sec via a digital stimulator (Digitimer Ltd, Hertfordshire, UK). Aδ-fiber EPSCs were distinguished from C-fiber EPSCs by the differences in latency to evoke an EPSC with C-fiber EPSC’s having longer latency than Aδ-fiber EPSC’s. Only monosynaptic evoked excitatory post synaptic currents (EPSC’s) were recorded. Perfusion to the spinal cord section also contained 50 μM picrotoxin and 50 μM strychnine to record only AMPA mediated EPSCs. Membrane signals were filtered at 2 kHz and sampled at 33 kHz.

### Synaptosome preparation and CGRP release assay

Male adult CD1 mice were terminally euthanized as described above, the spinal column removed and submerged in ice-cold ACSF bubbled with a 95/5% O_2_/CO_2_. A lumbosacral laminectomy was performed and a segment of lumbar spinal cord was removed into a petri dish containing ice-cold pre-oxygenated aCSF. The dura mater, ventral and dorsal roots and the pia-arachnoid membrane were removed. A small segment of the spinal cord containing only the lumbar region (L4–L5) was dissected free, weighed and placed into ice-cold homogenization buffer composition (mM) 0.32 M sucrose, 10 mM HEPES (pH 7.4, bubbled with 100% O_2_). Spinal cord tissue was homogenized in buffer using 20 strokes of a glass/glass hand homogenizer. The crude homogenate was diluted with 3 mL of ice-cold homogenization buffer and centrifuged at 1000 *x g* for 5 mins at 4°C. Synaptosomes were isolated from the supernatant by an additional centrifugation at 20,000 *x g* for 2 mins at 4°C. The synaptosomal pellet was resuspended in 3 mL of Krebs buffer with composition (in mM): NaCl 118, KCl 2.4, CaCl_2_ 2.4, MgSO_4_ 1.2, KH_2_PO_4_ 1.2, NaHCO_3_ 25, glucose 10, pH 7.4 (bubbled with 95% O_2_ and 5% CO_2_). Protein quantification was performed on the synaptosomal solution using the Bradford colorimetric protein assay and bovine serum albumin standards. Samples were diluted to obtain a final concentration of 0.1 mg mL^-1^ for CGRP evaluation and 100 μL samples of the synaptosomal solution was pipetted into eppendorfs. Synaptosomes were incubated at 37°C for 10 mins in the presence of test compounds or DMSO (1 μl total volume). Synaptosomes were stimulated using Veratridine 10 μM in the presence of Thiorphan (10 μM) and incubated for a further 10 mins at 37°C. Samples were then filtered using 0.22 μM filters and 100 μl of the filtrate was transferred to CGRP ELISA plate (Bertin Pharma) and assayed according to the manufactures instructions. Absorbance values were obtained using a microplate reader (Biorad model 680) at a wavelength of 415 nm.

### Capsaicin flare

Adult Male C57Bl/6J Wild type (WT) and Na_v_1.7Na_v_^1.8Cre^ mice were kept under a 12h light/dark cycle (lights on at 07:00) with food and water ad libitum. Up to 24h prior to dosing mice were shaved on both flanks and returned to their home cages. Subsequently, mice were grouped randomly and dosed orally with 1 or 10 mg/kg PF-05198007 or vehicle as appropriate (n = 8 per group). At 1h 15mins post dose mice were placed in an anesthetic chamber and anesthetized with a 5% isoflurane/O_2_ mixture. Anesthesia was maintained using a nose cone and animals transferred to a homeothermic blanket for the duration of the procedure. Laser Doppler flowmetry scans (MoorLDI apparatus, Moor Instruments Ltd, Axminster, United Kingdom) were taken from an area of the flank approximately 1.6cm^2^. Baseline scans were recorded for 35 mins, following which 50 μl of a 0.1% capsaicin (Sigma) dissolved in 100% ethanol was administered topically to the centre of the flank scan area using a 12mm polypropylene coated aluminium Finn chamber on Scanpor tape (Biodiagnostics Ltd, Worcestershire) for 10 min. Laser Doppler flowmetry scans were recorded for 55 mins post-removal of the Finn chamber. Laser Doppler flowmetry data was analyzed using proprietary software (MoorLDI, version 5.2). Baseline blood flow was calculated as the mean of the final 5 scans prior to capsaicin application.

### Data analysis

Patch clamp electrophysiological data analyses were performed using either pClamp v10 software (Molecular Devices), PatchMaster software (HEKA) or Spike 2 v7 (CED). Action potential parameters (voltage threshold, peak amplitude, upstroke slope, width at threshold) were determined using a bespoke Spike 2 v7 analysis script courtesy of CED. Data are presented as mean ± standard error of the mean (SEM) Statistical comparisons were made using the paired Student’s t-test, one-way analysis of variance (ANOVA) with Bonferroni or Tukey’s post-tests, or ANOVA on ranks.

### Drugs and reagents

PF-05089771 4-[2-(5-amino-1H-pyrazol-4-yl)-4-chlorophenoxy]-5-chloro-2-fluoro-N-(1,3-thiazol-4-yl)benzenesulfonamide and PF-05198007 [4-(2-(3-amino-1H-pyrazol-4-yl)-4-(trifluoromethyl)phenoxy)-5-chloro-2-fluoro-N-(thiazol-4-yl)benzenesulfonamide] were synthesized by Worldwide Medical Chemistry, Pfizer Worldwide Research & Development. Drugs were made up in 100% DMSO as a 10 mM stock, except for tetrodotoxin (TTX, Nanning Leaf Pharmaceuticals, Canada) which was dissolved in water. Final working concentrations were made on the day of the experiments in ECS.

## Results

### Discovery, potency and selectivity of PF-05089771 and PF-05198007

**Fig 1:**
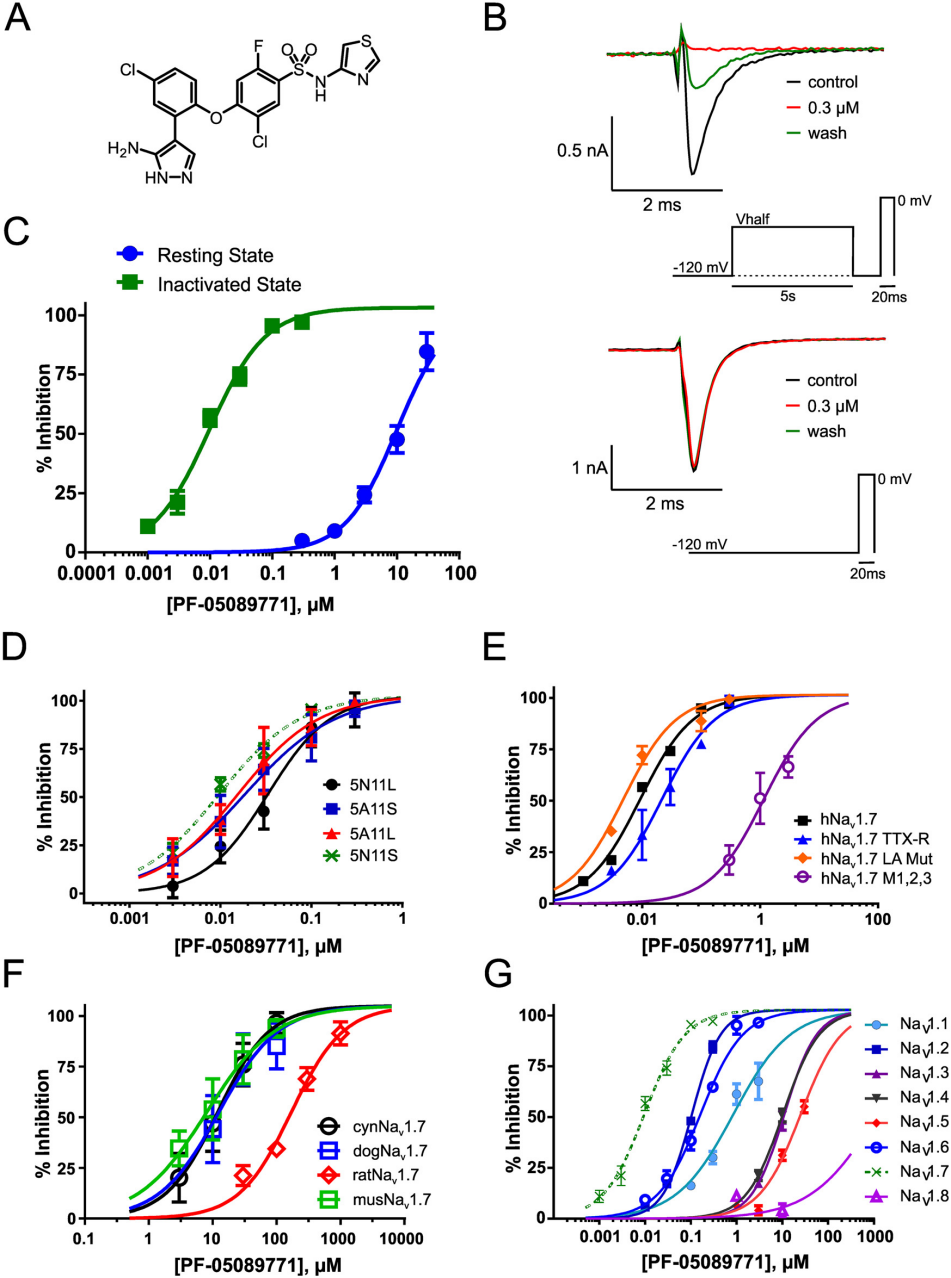
PF-05089771 is a potent, state-dependent and selective inhibitor of Na_v_1.7. A. Structure of PF-05089771 (4-(2-(3-amino-1H-pyrazol-4-yl)-4-chlorophenoxy)-5-chloro-2-fluoro-N-(thiazol-4-yl)benzenesulfonamide) B. Representative PatchXpress current recordings illustrating the near-complete block following 300 nM PF-05089771 application to half-inactivated WT hNa_v_1.7 channels (97% ± 3%, n = 10) which was partially reversed following a 5 min washout duration. In contrast there was minimal block following application of 300 nM PF-05089771 to resting WT hNa_v_1.7 channels (5% ± 3%, n = 4). Inset: PatchXpress voltage protocols for half-inactivation (upper) and resting state (lower). For a full description of the voltage protocols see Methods. C. Block of half-inactivated WT hNa_v_1.7 channels (n = 6–22 per concentration) was nearly 1000-fold more potent than resting channels (n = 4–11 per concentration) (11 nM *vs* 10 μM). D. Potency of PF-05089771 was similar across hNa_v_1.7 splice variants. IC_50_ values and Hill slopes are provided in Table 1. Data points represent n = 2–9 observations per concentration. E. PF-05089771 activity is impacted by mutation of a novel interaction site and not by local anaesthetic or toxin binding sites. Data points represent n = 3–6 observations per concentration except for hNa_v_1.7 where n = 6–22 observations per concentration. F. Potency of PF-05089771 was assessed on orthologous channels cloned from common preclinical species. IC_50_ values and Hill slopes are provided in Table 2. Data points represent n = 2–28 observations per concentration. G. PF-05089771 is a selective Na_v_1.7 subtype-selective inhibitor. Selectivity was assessed across a collection of heterologously expressed human Na_v_s on PatchXpress at the unique half inactivation voltage for each channel. Hill slopes and IC_50_ values are provided in Table 3. Data points represent n = 3–12 observations per concentration except for hNa_v_1.7 where n = 6–22 observations per concentration. Selectivity over the TTX-R Na_v_1.5 and Na_v_1.8 channels was greater than 1000-fold.

The identification of the clinical compound PF-05089771 and the closely related preclinical compound PF-05198007 as potent and selective inhibitors of the human Na_v_1.7 (hNa_v_1.7) channel resulted from iterative structure activity relationship—based refinement of a series of novel arylsulfonamide Na_v_ channel inhibitors. Arylsulfonamide inhibitors of Na_v_1.7 (as exemplified by PF-05089771, Fig 1A) were identified as inhibitors of partially inactivated hNa_v_1.7 channels by electrophysiological testing on the PatchXpress (Fig 1B and 1C) with a voltage protocol that set the holding potential to the empirically determined half-inactivation voltage for each cell (Fig 1B). For hNa_v_1.7 in HEK 293 cells the V_1/2_ of inactivation ranged from -54 mV to -96 mV with a mean value of -77.7 mV (n = 291). State-dependence of block was likewise assessed. In order to measure compound block at resting state, the original voltage protocol was modified to remove the conditioning step to the half-inactivation voltage, retaining all other aspects of the drug addition and voltage-control parameters (Fig 1B). Under these conditions the concentration-response relationship revealed an IC_50_ of 11 nM for half-inactivated channels and an IC_50_ of ~10 μM for resting channels, nearly 1000-fold less potent than seen with half-inactivated channels (Fig 1B and 1C).

Na_v_1.7 undergoes two independent alternative splicing events leading to the production of four splice isoforms of human Na_v_1.7 [19]. Implementing similar protocols as described above, PF-05089771 blocked all Na_v_1.7 splice variants with similar potency (Fig 1D, Table 1). Evaluation of the blockade of more distantly related channels, specifically, L-type calcium channels, KvLQT and hERG potassium channels, demonstrated that PF-05089771 is highly selective over these channels with IC_50_ values determined to be 10 μM. No blockade of the human and cynomolgus TRPV1 receptors was observed at tested concentrations of PF-05089771 up to 20 and 10 μM, respectively. (S1 Table).

**Table 1:** Potency of PF-05089771 across hNa_v_1.7 splice variants.

	5N11L	5A11S	5A11L	5N11S
IC_50_ (nM)	33	20	16	11
HillSlope	1.4	0.8	0.9	1.0
SEM	1.1	1.2	1.2	1.1

To examine the molecular basis of the Na_v_1.7 interaction with this novel inhibitor we measured PF-05089771 inhibition of channels that were mutated to change (1) the natural molecular determinant for TTX sensitivity in domain 1 (hNa_v_1.7 TTX-R, Y362S) [20], (2) local anesthetic binding residues (hNa_v_1.7 LA Mut, F1737A/Y1744A) [21] and (3) a recently identified small molecule binding site in VSD4 (hNa_v_1.7 M1,2,3 Y1537S/W1538R/D1586E) [22]. Data in Fig 1E show that the potency of PF-05089771 was not substantially affected (<3-fold) by mutation of either the TTX or local anesthetic binding sites hNa_v_1.7 (IC_50_ 11 ± 1.3 nM); hNa_v_1.7 TTX-R, (IC_50_ 21.6 ± 1.2 nM); hNa_v_1.7 LA Mut, (IC_50_ 4.7 ± 1.2 nM). However, PF-05089771 potency was reduced ~100 fold (IC_50_ 1.15 ± 1.2 μM) by the hNa_v_1.7 M1,2,3 mutation, indicating interaction of PF-05089771 with the domain 4 VSD (VSD4).

Based on differences in the cross species sequence homology at the VSD4 binding site for PF-05089771, the possibility of species-dependent changes in potency was investigated. The Na_v_1.7 orthologues were cloned from rat, mouse, dog and cynomologus macaque and each was heterologously expressed in HEK 293 cells to facilitate the cross species comparison. PF-05089771 was found to inhibit the sodium currents of mouse, dog and cynomologus macaque Na_v_1.7 with potency indistinguishable from that of the human channel (Table 2). However, the rat Na_v_1.7 potency was significantly decreased (IC_50_ 168 nM, 15.3-fold lower than human) compared to all other tested species, consistent with sequence divergence of the putative PF-05089771 binding residues within the VSD4 in rat Na_v_1.7 (H1547/W1548R/E1595, Fig 1F, Table 2) [22].

**Table 2:** Potency of PF-05089771 assessed at orthologous channels from selected species.

	cynNa_v_1.7	dogNa_v_1.7	ratNa_v_1.7	musNa_v_1.7
Slope	1.1	0.9	1.1	0.8
IC_50_ (nM)	12	13	171	8
IC_50_ SEM (nM)	4.9	5.3	1.2	1.2

Na_v_ channel isoform selectivity was also assessed using the half-inactivation voltage for each cell in hNa_v_1.x channels stably expressed in HEK293 cells. PF-05089771was determined to be more than 1000-fold selective over tetrodotoxin-resistant (TTX-R) Na_v_1.5 and Na_v_1.8 channels (IC_50_s >10 μM) and exhibited a range of selectivity over TTX-sensitive (TTX-S) channels (10-fold for Na_v_1.2 to 900-fold for Na_v_1.3 and Na_v_1.4) (Fig 1G, Table 3). Subsequently, these highly potent and selective Na_v_1.7 inhibitors permitted us to interrogate Na_v_1.7 in physiological preparations without pharmacological interference with other Na_v_ channel isoforms.

**Table 3:** Activity of PF-05089771 at human voltage-gated sodium channel isoforms.

	Na_v_1.1	Na_v_1.2	Na_v_1.3	Na_v_1.4	Na_v_1.5	Na_v_1.6	Na_v_1.7	Na_v_1.8
Slope	0.7	1.4	1.2	1.1	1.0	0.9	1.0	
IC_50_(μM)	0.85	0.11	11	10	25	0.16	0.011	> 10
SEM	1.2	1.1	1.2	1.2	1.1	1.2	1.3	

### Na_v_1.7 is the major TTX-sensitive Na_v_ channel in small-diameter DRG neurons

**Fig 2:**
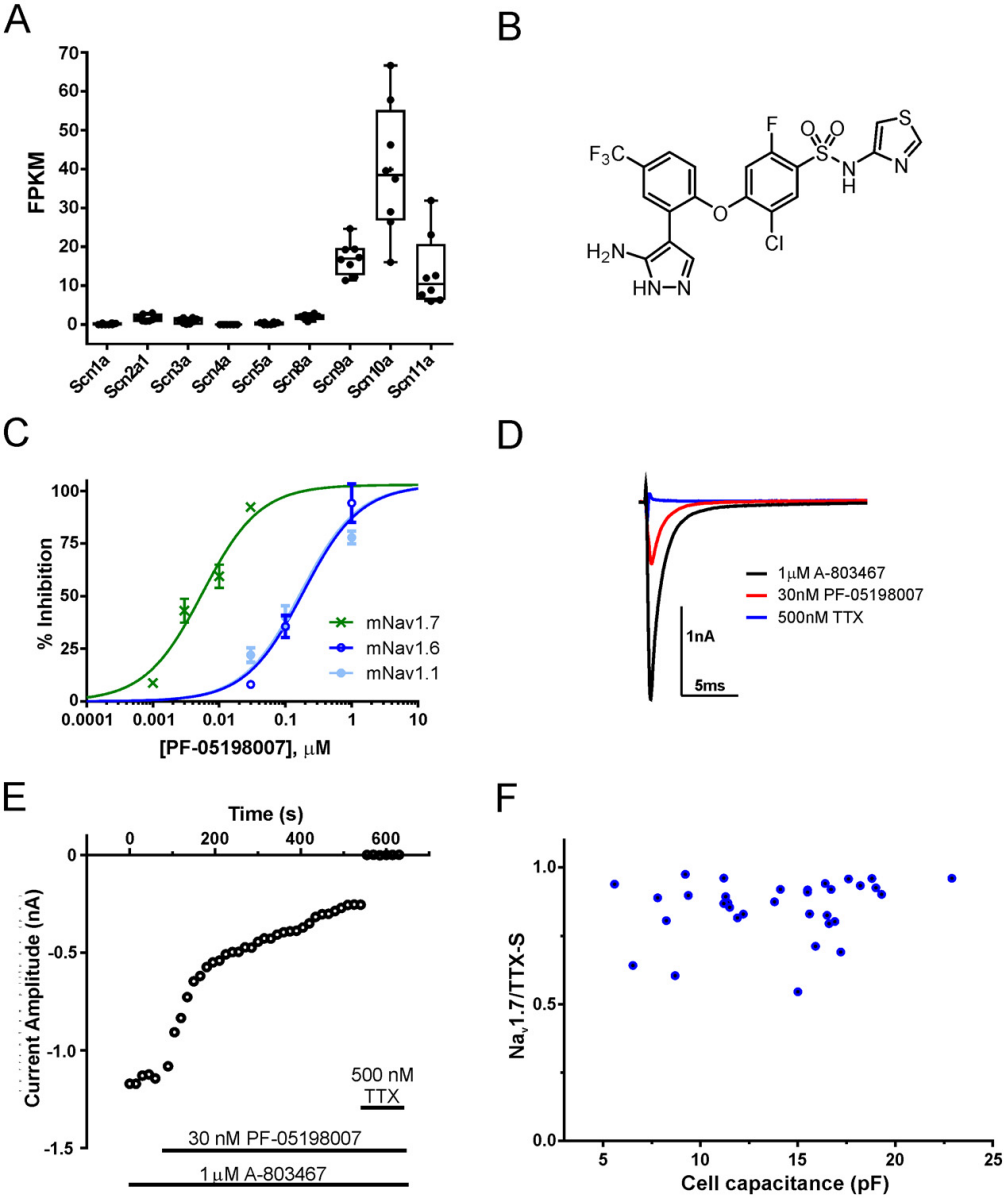
Na_v_1.7 is the major TTX-sensitive Na_v_ channel in small diameter mDRG neurons. A. RNASeq analysis of Na_v_ channel mRNA from pooled small diameter mouse DRG neurons. B. Structure of PF-05198007 (4-(2-(3-amino-1H-pyrazol-4-yl)-4-(trifluoromethyl)phenoxy)-5-chloro-2-fluoro-N-(thiazol-4-yl)benzenesulfonamide C. Patch clamp data showing concentration-response relationship for PF-05198007 assessed against recombinantly expressed mouse Na_v_1.7, Na_v_1.6 and Na_v_1.1 (IC_50_, Slope: 5.2 nM, 1.1; 149 nM, 1.5; 174 nM, 0.7 respectively; n = 3–4 per concentration). D. Representative patch clamp current traces of peak sodium current from small diameter mouse DRG neurons in the presence of A-803467 and following concurrent application of PF-05198007 and TTX. E. Representative peak TTX-S current *vs* time plot before and after 30 nM PF-05198007 and 500 nM TTX. G. Scatter plot of cell capacitance *vs* Na_v_1.7/TTX-S ratio (n = 35). Note that in every cell tested, Na_v_1.7 provided the predominant TTX-S sodium conductance.

To investigate the relative abundance of Na_v_1.7 in nociceptors,we examined the Na_v_ repertoire at the mRNA level in acutely dissociated small-diameter mouse DRG neurons using whole-transcriptome RNA sequencing. Fig 2A shows significant expression of mRNA for Na_v_1.7, Na_v_1.8 and Na_v_1.9 suggesting that Na_v_1.7 is the predominant TTX-S isoform. The functional influence of Na_v_1.7 compared to other TTX-S Na_v_ subtypes present in mouse DRG neurons was then assessed. For these experiments, PF-05198007 was used—a preclinical tool compound with an identical pharmacological selectivity profile (and marginal potency differences) to those described for PF-05089771, (Fig 2B and 2C) including slow kinetics of block which was fully reversible (using a resting-state protocol) after 20–30 min. Using whole-cell patch-clamp, the TTX-S current in small diameter mDRG neurons was isolated by application of the Na_v_1.8 blocker A-803467 (1 μM) [23]. Of the total Na^+^ current, an average of 34.1 ± 3.0% was blocked by A-803467 and the remaining current was abolished by TTX (i.e. the TTX-S current, n = 41). Application of PF-05198007 (30 nM) blocked on average 83.0 ± 2.7% of the total TTX-S current indicating that the major TTX-S conductance is carried through Na_v_1.7 channels in small-diameter mouse DRG neurons (n = 35, Fig 2D, 2E and 2F).

### Na_v_1.7 contributes to action potential threshold and upstroke in small mDRG neurons

**Fig 3:**
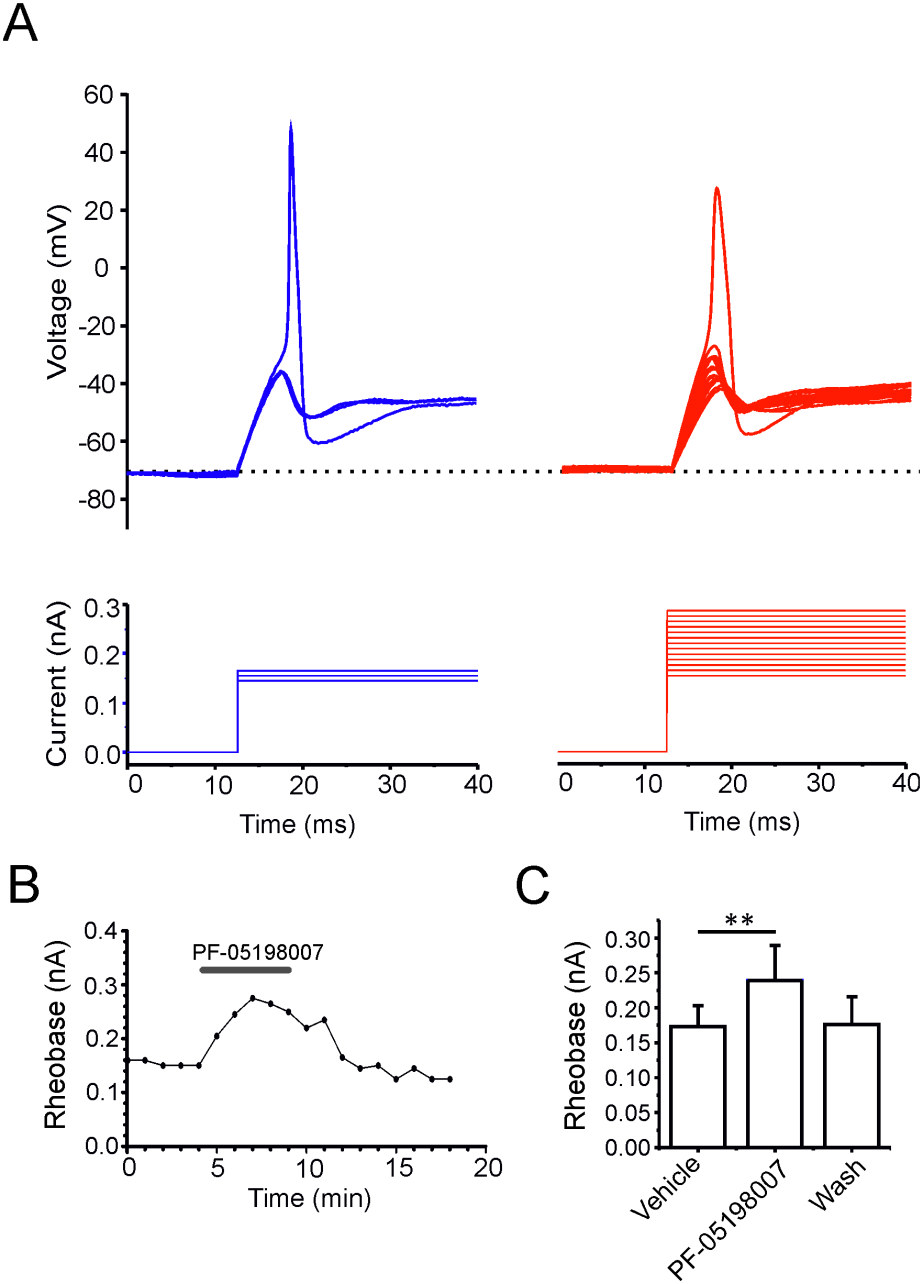
PF-05198007 increases action potential rheobase in small diameter mDRG neurons. A. Overlayed representative voltage traces in response to graded current step injections before (blue) and after PF-05198007 application (red). Current step stimulations are shown below. B. Example timecourse of change in rheobase following PF-05198007 application and washout. C. Summary bar graph, n = 8 neurons, ** p < 0.01, ANOVA. Data are shown ±SEM.

**Fig 4:**
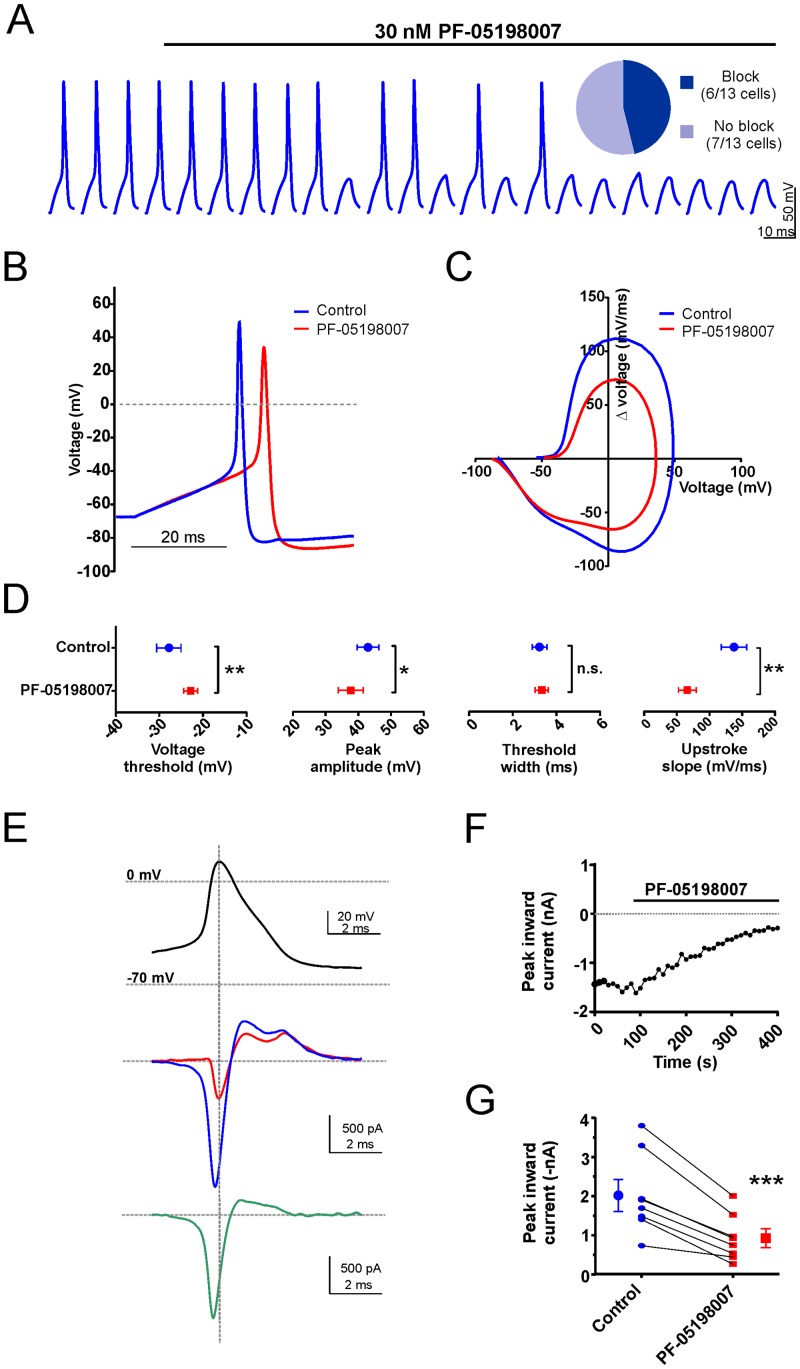
Na_v_1.7 controls action potential threshold and contributes to the rising phase. A. Example current clamp voltage traces in which PF-05198007 application resulted in action potential block. Single action potentials were evoked by a 20 ms suprathreshold current step at 0.1 Hz. The scale bar refers to the voltage traces whereas the start-to-start interval is 10 s. Inset: pie chart showing PF-05198007 (30 nM) resulted in complete action potential block in 6/13 neurons tested. B. Overlayed example action potential traces recorded from a neuron that exhibited partial block in response to PF-05198007 application. C. Phase plot of the action potential depicted in *B*. D. PF-05198007 depolarized the action potential voltage threshold and decreased both the peak amplitude and upstroke slope (n = 7 neurons, * p < 0.05, ** p < 0.01, paired t-test, data shown ±SEM). E. Example traces from an action potential voltage clamp experiment. Upper: the command action potential. Middle: overlayed current traces before (blue) and after PF-05198007 (30 nM, red). Lower: current subtraction revealing the current sensitive to block by PF-05198007. F. Example time-course of PF-05198007 effects on peak inward current. G. Scatter plot showing peak inward current for individual recordings as well as mathematical mean ± SEM before and after PF-05198007, n = 8 neurons, *** p < 0.001, paired t-test.

Previous studies have suggested that Na_v_1.7 contributes to electrogenesis by increasing the probability that the cell will reach action potential threshold [24] [20] [25]. To investigate this, current-clamp recordings were made from acutely dissociated mDRG neurons in which a series of sequentially larger current steps was applied in order to accurately determine the action potential rheobase. PF-05198007 significantly increased rheobase (control: 173 ± 37 pA *vs* PF-05198007: 239 ± 47 pA, n = 7, p < 0.01, ANOVA, Fig 3), an effect that returned to the control level following washout (control: 173 ± 37 pA *vs* wash: 176 ± 35 pA, p > 0.05, ANOVA, Fig 3B and 3C). The effect of PF-05198007 on rheobase confirms that Na_v_1.7 is important in setting action potential threshold in small-diameter DRG neurons. Subsequently, the effect of PF-05198007 on additional parameters of the action potential waveform was investigated. In current-clamp mode, single action potentials were evoked from -70 mV at 10 second intervals by 20 ms current steps to approximately 1.3 x rheobase. The aim of this protocol was to provide reliable action potential generation whilst minimizing any contaminating influence of the current step to the action potential waveform. In 6/13 small-diameter DRG neurons tested, PF-05198007 (30 nM) application resulted in action potential failure (Fig 4A) with the remaining 7 cells continued to exhibit action potential firing in the presence of PF-05198007. In the case of the latter, PF-05198007 impacted the action potential waveform whereby action potentials appeared delayed and were of smaller amplitude relative to control (Fig 4B). The representative phase plot in Fig 4C reveals the extent of waveform attenuation in the presence of PF-05198007. In particular, there was a reduction in the rate of voltage change during both the upstroke and repolarization phases, coupled with a reduction in the peak amplitude. PF-05198007 application resulted in a significant depolarization of action potential threshold (control: -27.8 ± 2.0 mV *vs* PF-05198007: -22.8 ± 1.6 mV, n = 10, p < 0.01) and a reduction in both spike amplitude (control: 43.0 ± 3.3 mV *vs* PF-05198007: 37.7 ± 3.9 mV, n = 10, p < 0.05) and upstroke slope (control: 137 ± 19 mV/ms *vs* PF-05198007: 86 ± 11 mV/ms, n = 10, p < 0.01, Fig 4D). In addition to confirming an important contribution to action potential threshold, these findings might suggest that Na_v_1.7 also contributes to the action potential upstroke phase. Alternatively, the changes in voltage trajectory that accompany a loss of threshold current might affect subsequent non-Na_v_1.7 sodium channel conductances so that the effects of PF-05198007 on amplitude and upstroke slope occur as an indirect consequence of Na_v_1.7 block. We tested this hypothesis by performing voltage-clamp experiments on mDRG neurons in which an action potential recorded from the cell under study was used as the command voltage (action potential voltage-clamp; Blair and Bean, 2002). The inward current component of the overlayed traces in Fig 4E (middle panel) shows an increased latency following PF-05198007 application accompanied by a decrease in the peak amplitude (in both cases p < 0.05, n = 7, Fig 4E, 4F and 4G). The subtraction of the residual current in the presence of PF-05198007 from the control current (Fig 4E lower panel) reveals an inward current that approximates to the Na_v_1.7 current flowing during the action potential. Comparison of this current time-course to that of the the action potential waveform illustrates that the peak of the PF-05198007-sensitive current occurred towards the peak of the action potential rising phase, suggesting a role for Na_v_1.7 not only in defining threshold but also in the action potential rising phase.

### The role of Na_v_1.7 in human DRG neurons

**Fig 5:**
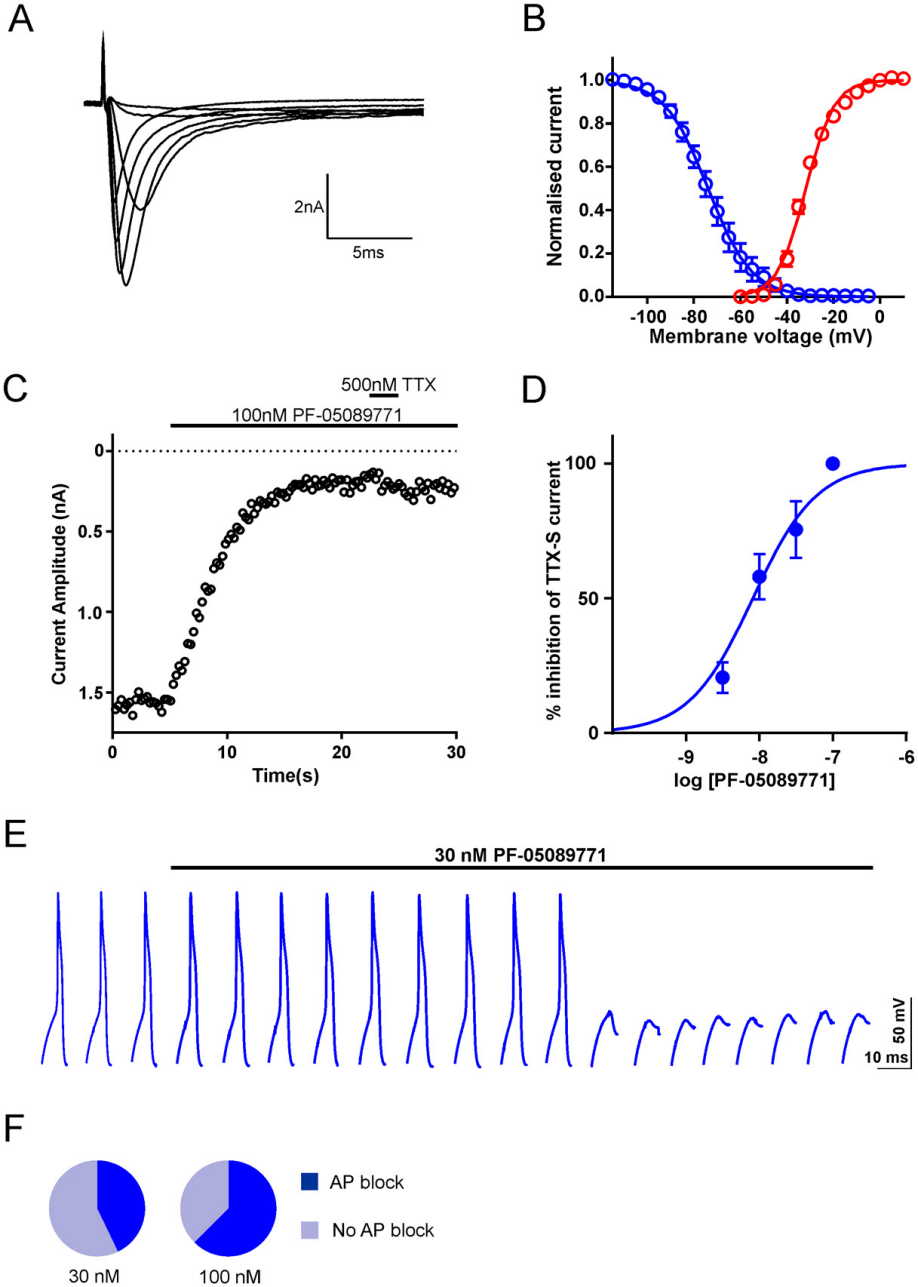
Evidence for functional Na_v_1.7 in human DRG neurons. A. Representative TTX-S current traces (recorded in the presence of 1 μM A-803467 and following graded voltage steps from -110 mV to 10 mV. B. Voltage dependence of activation (red, n = 4 for each voltage) generated from the protocol described in A and steady state fast inactivation (blue) generated by conditioning 500 msec prepulses to voltages between -110 mV and +10 mV followed by a test pulse to 0 mV from a holding potential of -110 mV (n = 4 for each voltage). Both datasets are fitted with Boltzmann functions. C. Representative timecourse relationship for peak TTX-S current following the application of 100 nM PF-05089771 and 500 nM TTX. D. Concentration-response relationship for PF-05089771 block of TTX-S current (IC_50_, slope: 8.4 nM, 1.1; n = 3–6 per concentration) E. Example voltage traces from a current clamp recording. Single action potentials were evoked by a 20 ms suprathreshold current step at 0.1 Hz. The scale bar refers to the voltage traces whereas the start-to-start interval is 10 s. F. Summary pie charts showing that the application of 30 and 100 nM PF-05089771 resulted in action potential block in 3/7 and 5/8 DRG neurons respectively.

To test whether our findings from isolated mouse DRG neurons extend to those derived from humans, electrophysiological recordings were made from small-to-medium diameter (<60 μm) DRG neurons surgically resected from human donors (hDRG neurons) and maintained in cell culture for up to 9 days. A recent study has reported that the majority of this cell population exhibit hallmarks of nociceptors [16]. Fast-inactivating TTX-S sodium currents were observed in all hDRG neurons (n = 16, Fig 5A). For these currents, the midpoint of the voltage activation and inactivation was –32.2 ± 0.8 mV and -71 ± 1.7 mV respectively (Fig 5B) compared with -22.0 ± 1.7 mV and -73.4 ± 1.5 mV recorded from human Na_v_1.7 recombinantly expressed in HEK 293 cells (p < 0.05; n = 9–10, data not shown). To assess the contribution of Na_v_1.7 in human DRG neurons directly, TTX-S currents were again isolated using A-803467 and recorded before and after application of PF-05089771. At a selective concentration (30 nM), this Na_v_1.7 inhibitor blocked the majority of TTX-S current (75.5 ± 10.5%, n = 5, Fig 5D) whilst 100 nM resulted in complete block (Fig 5C and 5D). The IC_50_ for TTX-S current block in hDRG neurons was 8.4 ± 1.2 nM (n = 5–8 per concentration) *vs* 11 ± 1.2 nM for recombinantly expressed hNa_v_1.7. Together, these data confirm that Na_v_1.7 underlies the majority of TTX-S current in cultured nociceptive hDRG neurons. We next explored how Na_v_1.7 contributes to electrogenesis in these cells. Fig 5E shows a voltage trace from a current-clamp recording in which we utilised the same current stimulus protocols as described for mDRG neurons (i.e. action potentials were evoked once every 10 seconds by 20 ms depolarizing current steps of amplitude just exceeding rheobase with the prestimulus V_m_ maintained at -70 mV). Under these recording conditions 30 nM PF-05089771 blocked action potentials in 3 out of 7 neurons and 100 nM resulted in action potential failure in 5 out of 8 cells tested (Fig 5E and 5F).

### Contribution of Na_v_1.7 to nociceptor signalling in the spinal cord

**Fig 6:**
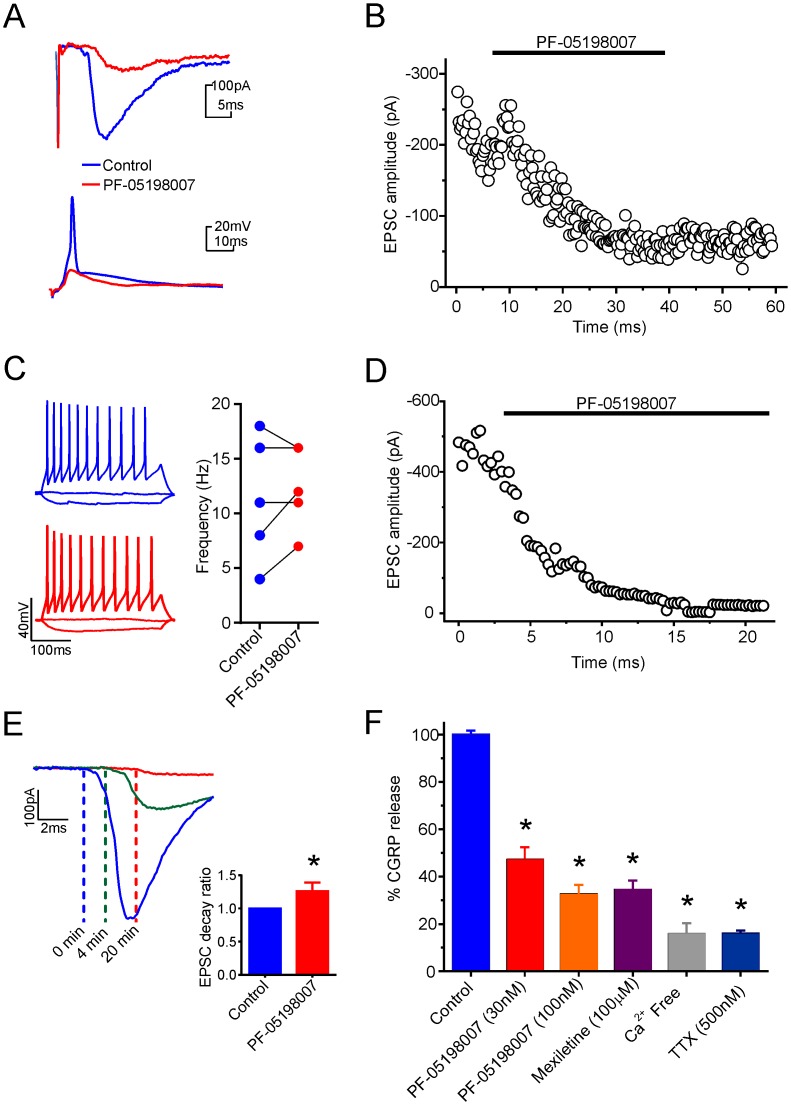
PF-05198007 acts peripherally and centrally to influence neurotransmitter release. A. Upper: representative evoked EPSCs during control (blue) and after 30 mins PF-05198007 application (red). Lower: representative synaptically evoked action potential trace (blue) recorded in SG neurons of the dorsal horn following dorsal root stimulation. PF-05198007 (20 mins) abolished synaptically evoked action potentials (red). B. Example time course of EPSC block following PF-05198007 application to the whole preparation. C. Action potentials induced *via* current injection steps in SG neurons were not abolished by PF-05198007 (30 nM). Representative voltage traces are shown following current injection steps of -20, 0 and 50 pA before (blue traces) and after (red traces) PF-05198007 application. Line chart shows change in firing frequency (Hz) during control and after application of PF-05198007 for all neurons tested (n = 5, p > 0.05, paired t-test). D. Example time course of EPSC block following PF-05198007 application to the dorsal root alone. E. Representative EPSC traces and summary bar graph showing that the application of PF-05198007 (30 nM) to the dorsal root alone inhibited C-fibre mediated EPSCs and resulted in a significant conduction delay (n = 7, * p < 0.05; ANOVA on Ranks). F. PF-05198007 (30 nM; n = 15: 100 nM; n = 19) reduced veratridine evoked CGRP release in spinal cord synaptosomes. Reduction was compared with mexilitine (100 μM; n = 19), Ca^2+^ free conditions (n = 8) and TTX (500 nM; n = 6) (Data are shown ±SEM; * p < 0.05; ANOVA on Ranks).

Within mouse spinal cord slices, stimulation of dorsal roots evoked both Aδ and C fiber excitatory post-synaptic currents (EPSCs) in substantia gelatinosa (SG) neurons of the superficial dorsal horn that were completely abolished by NBQX (5 μM) and TTX (500 nM; data not shown). Application of PF-05198007 to the whole preparation comprising spinal cord with attached dorsal root (30 nM; 20–30 mins) caused a significant inhibition of Aδ-fiber (by 46.9 ± 5.4%; n = 4, p < 0.05; ANOVA) and C-fiber (53.7 ± 10.1%; n = 8, p < 0.05; ANOVA, Fig 6A and 6B) evoked EPSC amplitudes. Under current-clamp conditions, electrical stimulation of the dorsal root evoked a single action potential in SG neurons. In the presence of 30 nM PF-05198007, such evoked action potentials were abolished in 8 out of 9 neurons tested (Fig 6A, lower trace). Action potentials were also evoked by injection of depolarizing current into SG neurons. Application of PF-05198007 (30 nM) had no effects on action potentials evoked in this manner (n = 6; Fig 6C) suggesting a presynaptic site of action for PF-05198007. To investigate the influence of axonally expressed Na_v_1.7 we used a dual perfusion bath that allowed PF-05198007 to be applied to the dorsal root specifically, without exposure to the spinal cord itself. Application of PF-05198007 (30 nM) to the dorsal root inhibited C-fiber evoked EPSCs by 87 ± 9% (n = 7; Fig 6D and 6E), and caused a significant (p < 0.05) delay in the latency of the C-fiber mediated EPSC (Fig 6E) suggesting a role for Na_v_1.7 in axonal action potential conduction.

To investigate the role of Na_v_1.7 in neuropeptide release from primary afferent central terminals we investigated the actions of PF-05198007 on veratridine-induced CGRP release from synaptosomes prepared from L4/L5 spinal cord sections (Fig 6F). Both PF-05198007 (30 nM & 100 nM) and mexiletine (100 μM) significantly reduced veratridine evoked CGRP release (in all cases p < 0.05; ANOVA on Ranks).

### PF-05198007 reduces the capsaicin flare response in WT, but not Na_v_1.7^Nav1.8Cre^ mice

**Fig 7:**
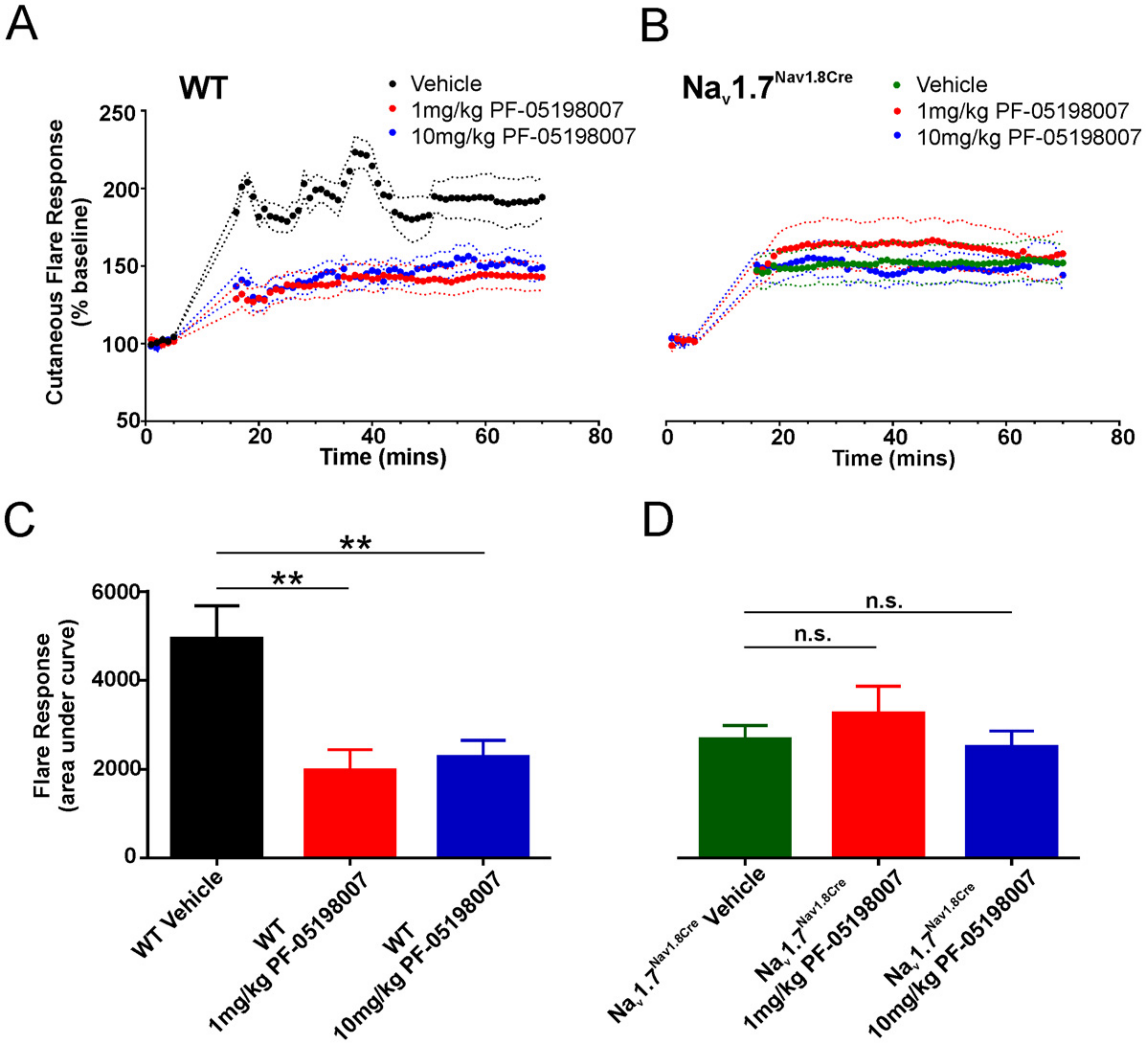
PF-05198007 reduces the capsaicin flare response in WT, but not Na_v_1.7^Nav1.8Cre^ mice. A, B.Time-course plots showing the effects of PF-05198007 on skin blood flow measured before and after topical capsaicin application for WT (A) and Nav1.7^Nav1.8Cre^ (B) mice (for each genotype, n = 8 per group). C, D. Corresponding summary bar graphs showing flare response measured as area under the curve for WT (C) and Nav1.7^Nav1.8Cre^ (D) mice before and after PF-05198007 treatment. 1 mg/kg and 10 mg/kg PF-05198007 significantly reduced capsaicin-induced flare in WT mice (C, both 1 mg/kg and 10 mg/kg, p < 0.01, ANOVA) but had no effect in Na_v_1.7^Nav1.8Cre^ mice (D, both 1 mg/kg and 10 mg/kg, p > 0.05, ANOVA).

To understand whether Na_v_1.7 contributes to neuropeptide release at the free nerve endings of nociceptors in the skin, in addition to CGRP release from central primary afferent terminals, we investigated the effects of the PF-05198007 on the capsaicin flare response *in vivo*. For WT mice, application of capsaicin (0.1%) induced a sustained flare response (Fig 7). Oral pre-treatment with the Na_v_1.7 inhibitor, PF-05198007 (1 and 10mg/kg) reduced the flare response to capsaicin for the duration of the observation period (55 mins; Vehicle; 4930 ± 751 versus 1 and 10 mg/kg 1967 ± 472 and 2265 ± 382, respectively (n = 7), AUC, p < 0.05, Fig 7A). To confirm that Na_v_1.7 block underlied this effect, we repeated the experiment using Na_v_1.7^Nav1.8 Cre^ mice in which Na_v_1.7 is genetically deleted only in Na_v_1.8-positive nocicetors (Nasser et al., 2004). Application of capsaicin to Na_v_1.7^Nav1.8 Cre^ mice resulted in a reduced (AUC, p < 0.01 *vs* WT, Fig 7B)—albeit still statistically significant—flare response (AUC, p < 0.05 *vs* pre-capsaicin baseline). Importantly, the flare response in Na_v_1.7 ^Nav1.8 Cre^ mice was indistinguishable from the residual flare response in WT PF-05198007-treated mice. PF-05198007 (1, 10 mg/kg), had no effect on the flare response in Na_v_1.7 ^Nav1.8 Cre^ mice (for both doses, p > 0.05, Fig 7B) indicating that the Na_v_1.7 component of flare in WT mice was completely blocked by the selective Na_v_1.7 inhibitor whilst other mechanisms that contribute to flare remained unaffected. These data demonstrate a Na_v_1.7-mediated contribution to neuropeptide release in peripheral neurogenic flare.

## Discussion

There recently has been intense interest in development of subtype-selective Na_v_1.7 inhibitors. This has arisen from the identification of gain-of-function mutations of Na_v_1.7 in individuals with rare pain syndromes IEM, PEPD and the more common disorder SFN, as well as the discovery of this channel’s link to channelopathy-associated congenital insensitivity to pain. Together, this constellation of gain-of-function and loss-of-function mutations demonstrates an essential and non-redundant role for Na_v_1.7 in pain [1]. Here we describe *in vitro* pharmacological properties of two subtype-selective Na_v_1.7 compounds: a clinical compound PF-05089771 and a structurally-related preclinical tool compound PF-05198007. Using these selective inhibitors we have confirmed a functional role of Na_v_1.7 that spans the sensory neuron axis—from the cutaneous free nerve endings, through to the DRG cell body, dorsal root and central terminals in the spinal cord.

### Pharmacological properties of Na_v_1.7 subtype-selective compounds

The potency of PF-05089771 against human Na_v_1.7 channels stably expressed in HEK293 cells (IC_50_, 11 nM) was 10-fold and 16-fold higher than the closest sodium channel isoforms Na_v_1.2 and Na_v_1.6, respectively. Other sodium channel subtypes that have been shown to play important roles in the pain pathways and that are expressed in DRG neurons, Na_v_1.3 and Na_v_1.8 [26], are essentially unaffected by PF-05089771. A lack of validated heterologous expression system for Na_v_1.9 prohibited evaluation of the potency of PF-05089771 against this channel, which has been shown to play an important role in human pain conditions [27]. The potency and selectivity of PF-05089771 have been achieved by targeting a novel binding region of Na_v_1.7 proposed to be in the VSD4 [22,28]. The interaction of PF-05089771 with this novel binding site is further supported by the inability of mutations altering the local anesthetic and TTX binding sites in Na_v_1.7 to significantly alter the potency of PF-05089771 examined *in vitro*. The selectivity gained through interacting with the channel at this site is consistent with the variability of critical amino acids across sodium channel isoforms, supporting a modulatory effect of PF-05089771 on the VSD of Na_v_1.7. Finally, this is also the case for several species orthologues of Na_v_1.7 where PF-05089771 was found to preferentially inhibit human, mouse, cynomolgus monkey and dog Na_v_1.7 over rat Na_v_1.7. The rat orthologue possesses two variant residues at the VSD4 interaction site and uniquely displays a histidine residue at position M1, differentiating rat from the other species that were tested. Thus, the unprecedented selectivity profile achieved by PF-05089771 and the cross-species differences in Na_v_1.7 orthologue potency can be explained through the novel binding interaction of this class of compounds.

### Na_v_1.7 dominates TTX-S current in small-diameter DRG neurons

Several lines of evidence have suggested that Na_v_1.7 is the predominant Na_v_ TTX-S subtype expressed in C-fibres, while Na_v_1.7, Na_v_1.6 and Na_v_1.1 are the main TTX-S subtypes present in A-fibres [29] [11] [26] [30] [12] [1] [13]. Using RNA-Seq to analyse pooled small-diameter mouse DRG neurons, we also demonstrate that Na_v_1.7 is the major TTX-S subtype transcript in nociceptors. We found that high Na_v_1.7 expression translates at the functional level because the majority of the isolated TTX-S current in the cell body of small-diameter mouse and human DRG neurons was blocked by Na_v_1.7 inhibitors. This data is consistent with published work showing that TTX-S current in small-diameter DRG neurons from the Na_v_1.7^Nav1.8 Cre^ mouse is reduced by 70% compared to WT [31]. Taken together, these findings imply that Na_v_1.7 is likely to play a prominent role in nociceptor excitability.

### The role of Na_v_1.7 in electrogenesis

Our electrophysiological studies demonstrate the important contribution of Na_v_1.7 to both mouse and human sensory neuron action potential firing. In particular, the effects of Na_v_1.7 inhibitors on mouse DRG rheobase provide the first pharmacological evidence using a small molecule selective inhibitor that native Na_v_1.7 acts as the threshold channel. These data are consistent with recorded changes in rheobase following Na_v_1.7 silencing in cultured nodose sensory neurons [32] and dynamic clamp analysis, using a Hodgkin-Huxley model of hNa_v_1.7 gating [31]. Furthermore our observation of a reduction in action potential peak height and slope in the presence of PF-05198007 suggests a role for Na_v_1.7 in the upstroke of the action potential, in addition to the more accepted idea of Na_v_1.7 as a threshold channel. Indeed, using the action potential voltage-clamp technique [33] we found that Na_v_1.7 conducts during the upstroke as well as the during initiation stage of the action potential. Although Na_v_1.7 has been suggested to amplify the generator potential at the peripheral terminals thus acting a s a threshold channel [24], we propose that another principal role for Na_v_1.7 in nociceptors is in action potential electrogenesis.

### Central and peripheral role of Na_v_1.7 in nociceptors

The role of Na_v_1.7 in synaptic transmission within the spinal cord was addressed using patch-clamp recordings from dorsal horn SG neurons following stimulation of the dorsal root. Evoked EPSCs were significantly reduced in amplitude by PF-05198007 perfused over the entire spinal cord slice preparation. These findings agree with previous reports demonstrating reduced Substance P release from spinal cord slices of Na_v_1.7 knockout mice (Na_v_1.7 ablated in advillin-expressing neurons) following electrical stimulation of attached dorsal root [34] and the reduced frequency of spontaneous EPSCs in mouse lamina II neurons following the application of a Na_v_1.7 blocking antibody [35]. In addition, a central role of Na_v_1.7 has been demonstrated *in vivo* where a reduction in pain behaviour in mice is observed following intrathecal injection of this antibody [35]. In this scenario, Na_v_1.7 block can occur at both dorsal root and outer laminae. Here, the specific contributions of Na_v_1.7 to both conduction and synaptic release in the spinal cord were further investigated using PF-5198007. Specifically, we observed a reduction in C-fibre mediated EPSC amplitudes recorded from SG neurons (with corresponding slowing of C-fibre conduction velocity) following local application of PF-05198007 to dorsal root, implicating Na_v_1.7 in axonal conduction. In support of this, significant block of A-and C-fibre action potentials in vagal sensory neurons has been reported following shRNA knockdown of Na_v_1.7 [32] and similar findings have been obtained from sciatic nerve in studies using toxins that selectively inhibit different Na_v_ subtypes including Na_v_1.7 [36] [37]. Collectively, these data are consistent with a role for Na_v_1.7 in which it influences synaptic transmission in the dorsal horn *via* actions either at a peripheral location (i.e. by facilitating or permitting conduction), or at a central location (i.e. by affecting CGRP/Substance P release from central terminals), or both.

TRPV1 expression has previously been shown to be limited to C-fibres in mouse [38] [39] [13]. Therefore, neurogenic flare induced by topical application of a non-noxious dose of capsaicin represents a C-fibre mediated mechanism in mouse. Using the Laser Doppler flowmetry to measure increased blood flow associated with peripherally mediated axon reflex vasodilation [40], capsaicin-induced neurogenic flare was suppressed by the Na_v_1.7 inhibitor PF-05198007 in wild-type mouse, but this inhibitor had no effect on the Na_v_1.7^Nav1.8Cre^ knock-out mouse at the same dose. These data suggest a key role of Na_v_1.7 in controlling neuropeptide release from peripheral terminals of peptidergic C-fibres and potentially a role in conduction associated with the axon reflex.

## Conclusion

The data in the current study could be interpreted as supporting separate roles for Na_v_1.7 in neurogenic flare, conduction and presynaptic release. However, peptidergic terminals in the dorsal horn consist of *en passant* varicosities [41] giving rise to simple non-glomerular axo-dendritic synapses [42]. Voltage-gated sodium channels have also been identified in *en passant* varicosities of both posterior pituitary and mossy fibre terminals, where they have been suggested to contribute to action potential generation and conduction [43] [44]. Therefore, a simple unifying theory, which takes into account the clear role of Na_v_1.7 in action potential generation at multiple sites, is that Na_v_1.7 contributes to electrogenesis at, or close to, distal terminals in peripheral axon, in the dorsal root and at, or close to, neurotransmitter-releasing boutons of nociceptors. The development and availability of selective Na_v_1.7 inhibitors will aid future studies to delineate the interplay between Na_v_1.7 function and nociceptor signalling in both acute and chronic pain states.

## Supporting Information

S1 Table Off target activity of PF-5089771. (DOCX)

## References

[1] Dib-Hajj SD, Yang Y, Black JA, Waxman SG (2013) The Na(V)1.7 sodium channel: from molecule to man. Nat Rev Neurosci 14: 49–62. 10.1038/nrn3404 23232607

[2] Bennett DL, Woods CG (2014) Painful and painless channelopathies. Lancet Neurol 13: 587–599. 10.1016/S1474-4422(14)70024-9 24813307

[3] Cox JJ, Reimann F, Nicholas AK, Thornton G, Roberts E, Springell K, et al (2006) An SCN9A channelopathy causes congenital inability to experience pain. Nature 444: 894–898. 17167479 10.1038/nature05413 PMC7212082

[4] Fertleman CR, Baker MD, Parker KA, Moffatt S, Elmslie FV, Abrahamsen B, et al (2006) SCN9A mutations in paroxysmal extreme pain disorder: allelic variants underlie distinct channel defects and phenotypes. Neuron 52: 767–774. 17145499 10.1016/j.neuron.2006.10.006

[5] Cummins TR, Dib-Hajj SD, Waxman SG (2004) Electrophysiological properties of mutant Nav1.7 sodium channels in a painful inherited neuropathy. J Neurosci 24: 8232–8236. 15385606 10.1523/JNEUROSCI.2695-04.2004 PMC6729696

[6] Yang Y, Wang Y, Li S, Xu Z, Li H, Ma L, et al (2004) Mutations in SCN9A, encoding a sodium channel alpha subunit, in patients with primary erythermalgia. J Med Genet 41: 171–174. 14985375 10.1136/jmg.2003.012153 PMC1735695

[7] Dib-Hajj SD, Rush AM, Cummins TR, Hisama FM, Novella S, Tyrrell L, et al (2005) Gain-of-function mutation in Nav1.7 in familial erythromelalgia induces bursting of sensory neurons. Brain 128: 1847–1854. 15958509 10.1093/brain/awh514

[8] Drenth JP, Waxman SG (2007) Mutations in sodium-channel gene SCN9A cause a spectrum of human genetic pain disorders. J Clin Invest 117: 3603–3609. 18060017 10.1172/JCI33297 PMC2096434

[9] Black JA, Frezel N, Dib-Hajj SD, Waxman SG (2012) Expression of Nav1.7 in DRG neurons extends from peripheral terminals in the skin to central preterminal branches and terminals in the dorsal horn. Mol Pain 8: 82 10.1186/1744-8069-8-82 23134641 PMC3517774

[10] Black JA, Dib-Hajj S, McNabola K, Jeste S, Rizzo MA, Kocsis JD, et al (1996) Spinal sensory neurons express multiple sodium channel alpha-subunit mRNAs. Brain Res Mol Brain Res 43: 117–131. 9037525 10.1016/s0169-328x(96)00163-5

[11] Fukuoka T, Kobayashi K, Yamanaka H, Obata K, Dai Y, Noguchi K (2008) Comparative study of the distribution of the alpha-subunits of voltage-gated sodium channels in normal and axotomized rat dorsal root ganglion neurons. J Comp Neurol 510: 188–206. 10.1002/cne.21786 18615542

[12] Ho C, O'Leary ME (2011) Single-cell analysis of sodium channel expression in dorsal root ganglion neurons. Mol Cell Neurosci 46: 159–166. 10.1016/j.mcn.2010.08.017 20816971 PMC3005531

[13] Usoskin D, Furlan A, Islam S, Abdo H, Lonnerberg P, Lou D, et al (2015) Unbiased classification of sensory neuron types by large-scale single-cell RNA sequencing. Nat Neurosci 18: 145–153. 10.1038/nn.3881 25420068

[14] Bagal SK, Chapman ML, Marron BE, Prime R, Storer RI, Swain NA (2014) Recent progress in sodium channel modulators for pain. Bioorg Med Chem Lett 24: 3690–3699. 10.1016/j.bmcl.2014.06.038 25060923

[15] Passmore GM (2005) Dorsal root ganglion neurones in culture: a model system for identifying novel analgesic targets? J Pharmacol Toxicol Methods 51: 201–208. 15862465 10.1016/j.vascn.2004.08.007

[16] Davidson S, Copits BA, Zhang J, Page G, Ghetti A, Gereau RWt (2014) Human sensory neurons: Membrane properties and sensitization by inflammatory mediators. Pain 155: 1861–1870. 10.1016/j.pain.2014.06.017 24973718 PMC4158027

[17] Pearce RJ, Duchen MR (1994) Differential expression of membrane currents in dissociated mouse primary sensory neurons. Neuroscience 63: 1041–1056. 7535391 10.1016/0306-4522(94)90571-1

[18] Castle N, Printzenhoff D, Zellmer S, Antonio B, Wickenden A, Silvia C (2009) Sodium channel inhibitor drug discovery using automated high throughput electrophysiology platforms. Comb Chem High Throughput Screen 12: 107–122. 19149496 10.2174/138620709787047993

[19] Raymond CK, Castle J, Garrett-Engele P, Armour CD, Kan Z, Tsinoremas N, et al (2004) Expression of alternatively spliced sodium channel alpha-subunit genes. Unique splicing patterns are observed in dorsal root ganglia. J Biol Chem 279: 46234–46241. 15302875 10.1074/jbc.M406387200

[20] Herzog RI, Cummins TR, Ghassemi F, Dib-Hajj SD, Waxman SG (2003) Distinct repriming and closed-state inactivation kinetics of Nav1.6 and Nav1.7 sodium channels in mouse spinal sensory neurons. J Physiol 551: 741–750. 12843211 10.1113/jphysiol.2003.047357 PMC2343279

[21] Ragsdale DS, McPhee JC, Scheuer T, Catterall WA (1996) Common molecular determinants of local anesthetic, antiarrhythmic, and anticonvulsant block of voltage-gated Na+ channels. Proc Natl Acad Sci U S A 93: 9270–9275. 8799190 10.1073/pnas.93.17.9270 PMC38631

[22] McCormack K, Santos S, Chapman ML, Krafte DS, Marron BE, West CW, et al (2013) Voltage sensor interaction site for selective small molecule inhibitors of voltage-gated sodium channels. Proc Natl Acad Sci U S A 110: E2724–2732. 10.1073/pnas.1220844110 23818614 PMC3718154

[23] Jarvis MF, Honore P, Shieh CC, Chapman M, Joshi S, Zhang XF, et al (2007) A-803467, a potent and selective Nav1.8 sodium channel blocker, attenuates neuropathic and inflammatory pain in the rat. Proc Natl Acad Sci U S A 104: 8520–8525. 17483457 10.1073/pnas.0611364104 PMC1895982

[24] Cummins TR, Howe JR, Waxman SG (1998) Slow closed-state inactivation: a novel mechanism underlying ramp currents in cells expressing the hNE/PN1 sodium channel. J Neurosci 18: 9607–9619. 9822722 10.1523/JNEUROSCI.18-23-09607.1998 PMC6793269

[25] Rush AM, Cummins TR, Waxman SG (2007) Multiple sodium channels and their roles in electrogenesis within dorsal root ganglion neurons. J Physiol 579: 1–14. 17158175 10.1113/jphysiol.2006.121483 PMC2075388

[26] Dib-Hajj SD, Cummins TR, Black JA, Waxman SG (2010) Sodium channels in normal and pathological pain. Annu Rev Neurosci 33: 325–347. 10.1146/annurev-neuro-060909-153234 20367448

[27] Dib-Hajj SD, Black JA, Waxman SG (2015) NaV1.9: a sodium channel linked to human pain. Nat Rev Neurosci 16: 511–519. 10.1038/nrn3977 26243570

[28] Ahuja S, Mukund S, Deng L, Khakh K, Chang E, Ho H, et al (2015) Structural basis of Nav1.7 inhibition by an isoform-selective small-molecule antagonist. Science 350: aac5464 10.1126/science.aac5464 26680203

[29] Djouhri L, Fang X, Okuse K, Wood JN, Berry CM, Lawson SN (2003) The TTX-resistant sodium channel Nav1.8 (SNS/PN3): expression and correlation with membrane properties in rat nociceptive primary afferent neurons. J Physiol 550: 739–752. 12794175 10.1113/jphysiol.2003.042127 PMC2343087

[30] Fukuoka T, Noguchi K (2011) Comparative study of voltage-gated sodium channel alpha-subunits in non-overlapping four neuronal populations in the rat dorsal root ganglion. Neurosci Res 70: 164–171. 10.1016/j.neures.2011.01.020 21303679

[31] Vasylyev DV, Han C, Zhao P, Dib-Hajj S, Waxman SG (2014) Dynamic-clamp analysis of wild-type human Nav1.7 and erythromelalgia mutant channel L858H. J Neurophysiol 111: 1429–1443. 10.1152/jn.00763.2013 24401712

[32] Muroi Y, Ru F, Kollarik M, Canning BJ, Hughes SA, Walsh S, et al (2011) Selective silencing of Na(V)1.7 decreases excitability and conduction in vagal sensory neurons. J Physiol 589: 5663–5676. 10.1113/jphysiol.2011.215384 22005676 PMC3249041

[33] Blair NT, Bean BP (2002) Roles of tetrodotoxin (TTX)-sensitive Na+ current, TTX-resistant Na+ current, and Ca2+ current in the action potentials of nociceptive sensory neurons. J Neurosci 22: 10277–10290. 12451128 10.1523/JNEUROSCI.22-23-10277.2002 PMC6758735

[34] Minett MS, Nassar MA, Clark AK, Passmore G, Dickenson AH, Wang F, et al (2012) Distinct Nav1.7-dependent pain sensations require different sets of sensory and sympathetic neurons. Nat Commun 3: 791 10.1038/ncomms1795 22531176 PMC3337979

[35] Lee JH, Park CK, Chen G, Han Q, Xie RG, Liu T, et al (2014) A monoclonal antibody that targets a NaV1.7 channel voltage sensor for pain and itch relief. Cell 157: 1393–1404. 10.1016/j.cell.2014.03.064 24856969 PMC4098795

[36] Schmalhofer WA, Calhoun J, Burrows R, Bailey T, Kohler MG, Weinglass AB, et al (2008) ProTx-II, a selective inhibitor of NaV1.7 sodium channels, blocks action potential propagation in nociceptors. Mol Pharmacol 74: 1476–1484. 10.1124/mol.108.047670 18728100

[37] Wilson MJ, Yoshikami D, Azam L, Gajewiak J, Olivera BM, Bulaj G, et al (2011) mu-Conotoxins that differentially block sodium channels NaV1.1 through 1.8 identify those responsible for action potentials in sciatic nerve. Proc Natl Acad Sci U S A 108: 10302–10307. 10.1073/pnas.1107027108 21652775 PMC3121861

[38] Cavanaugh DJ, Chesler AT, Braz JM, Shah NM, Julius D, Basbaum AI (2011) Restriction of transient receptor potential vanilloid-1 to the peptidergic subset of primary afferent neurons follows its developmental downregulation in nonpeptidergic neurons. J Neurosci 31: 10119–10127. 10.1523/JNEUROSCI.1299-11.2011 21752988 PMC3147010

[39] Lawson JJ, McIlwrath SL, Woodbury CJ, Davis BM, Koerber HR (2008) TRPV1 unlike TRPV2 is restricted to a subset of mechanically insensitive cutaneous nociceptors responding to heat. J Pain 9: 298–308. 10.1016/j.jpain.2007.12.001 18226966 PMC2372162

[40] Klede M, Handwerker HO, Schmelz M (2003) Central origin of secondary mechanical hyperalgesia. J Neurophysiol 90: 353–359. 12843313 10.1152/jn.01136.2002

[41] Nagy JI, Hunt SP (1983) The termination of primary afferents within the rat dorsal horn: evidence for rearrangement following capsaicin treatment. J Comp Neurol 218: 145–158. 6193151 10.1002/cne.902180203

[42] Ribeiro-da-Silva A, Tagari P, Cuello AC (1989) Morphological characterization of substance P-like immunoreactive glomeruli in the superficial dorsal horn of the rat spinal cord and trigeminal subnucleus caudalis: a quantitative study. J Comp Neurol 281: 497–415. 2468697 10.1002/cne.902810402

[43] Jackson MB, Zhang SJ (1995) Action potential propagation and propagation block by GABA in rat posterior pituitary nerve terminals. J Physiol 483 (Pt 3): 597–611. 7776246 10.1113/jphysiol.1995.sp020609 PMC1157805

[44] Engel D, Jonas P (2005) Presynaptic action potential amplification by voltage-gated Na+ channels in hippocampal mossy fiber boutons. Neuron 45: 405–417. 15694327 10.1016/j.neuron.2004.12.048

