# Supplementary material for: Subtype-Selective Small Molecule Inhibitors Reveal a Fundamental Role for Nav1.7 in Nociceptor Electrogenesis, Axonal Conduction and Presynaptic Release
Source: PLoS One. 2016 Apr 6;11(4):e0152405. doi: 10.1371/journal.pone.0152405 (PMC4822888; doi:10.1371/journal.pone.0152405)
Supplement: S1 Table — (DOCX) [file pone.0152405.s001.docx]

S1 Table

| **Assay** | **Test Concentration (**μ**M)** | **Inhibition at test concentration (%)** | **Ki (uM)** | **Functional activity** |
| --- | --- | --- | --- | --- |
| Adenosine A1 receptor | 10 | 15 |  |  |
| Adenosine A2a receptor | 10 | 15 |  |  |
| Adenosine A3 receptor | 10 | 4 |  |  |
| Abl kinase receptor | 10 | 9 |  |  |
| Angiotensin converting enzyme | 10 | -4 |  |  |
| Acetylcholinesterase | 10 | 17 |  |  |
| Adrenergic alpha 1 receptor | 10 | -5 |  |  |
| Adrenergic alpha 2A receptor | 10 | 10 |  |  |
| Adrenergic alpha 2B receptor | 10 | 24 |  |  |
| AMPA receptor | 10 | -13 |  |  |
| Androgen receptor | 10 | 2 |  |  |
| Beta 1 adrenergic receptor | 10 | 12 |  |  |
| Beta 2 adrenergic receptor | 10 | 4 |  |  |
| GABA Benzodiazepine site | 10 | 5 |  |  |
| Calcium channel L-type (dihydropyridine site) | 10 | 4 |  |  |
| Calcium channel L-type  (diltiazem site) | 10 | 16 |  |  |
| Calcium channel L-type  (verapamil site) | 10 | -6 |  |  |
| Cannabinoid receptor 1 | 10 | 0 |  |  |
| Cannabinoid receptor 2 | 10 | -5 |  |  |
| Carbonic anhydrase II | 10 | 21 |  |  |
| Choline transporter | 10 | 18 |  |  |
| Cloride channel (GABA-gated) | 10 | 54 | 8.3 |  |
| Cholycystokinin 1 (CCKA) receptor | 10 | 17 |  |  |
| Cholycystokinin 2 (CCKB) receptor | 10 | 13 |  |  |
| Cyclooxygenase 1 | 10 | 18 |  |  |
| Cyclooxegenase 2 | 10 | 72 | 5.4 |  |
| Delta opioid receptor | 10 | 15 |  |  |
| Dopamine D1 receptor | 10 | 0 |  |  |
| Dopamine D2 receptor | 10 | 10 |  |  |
| Dopamine D3 receptor | 10 | 16 |  |  |
| Dopamine D4 receptor | 10 | 5 |  |  |
| Dopamine transporter | 10 | 18 |  |  |
| FLT-1 kinase | 10 | 14 |  |  |
| GABA_A_ channel | 10 | 41 |  |  |
| GABA transporter | 10 | 1 |  |  |
| Glucocorticoid receptor | 10 | 3 |  |  |
| Histamine H1 receptor | 10 | 12 |  |  |
| Histamine H2 receptor | 10 | -3 |  |  |
| Histamine H3 receptor | 10 | 10 |  |  |
| Kappa opioid receptor | 10 | 86 | 0.39 | no agonist or antagonist activity at 10 μM |
| Lyn A kinase | 10 | 51 | 7.3 |  |
| Matrix metalloprotease 9 | 10 | -3 |  |  |
| Melanin-concentrating hormone receptor 1 | 10 | -7 |  |  |
| Melanocortin 4 receptor | 10 | -11 |  |  |
| Monoamine oxidase A | 10 | -13 |  |  |
| Metallothionein 3 (ML2) | 10 | 64 | 5.5 |  |
| Motilin receptor | 10 | 11 |  |  |
| Mu opiod receptor | 10 | 7 |  |  |
| Muscarinic M1 receptor | 10 | 11 |  |  |
| Muscarinic M2 receptor | 10 | 3 |  |  |
| Muscarinic M3 receptor | 10 | 16 |  |  |
| Muscle-type nicotinic receptor | 10 | 2 |  |  |
| Neurokinin NK1 receptor | 10 | 5 |  |  |
| Neurokinin NK2 receptor | 10 | -10 |  |  |
| Nictotinic acetylcholine receptor | 10 | -7 |  |  |
| NMDA receptor (phencyclidine site) | 10 | -3 |  |  |
| Norepinephrine transporter | 10 | 27 |  |  |
| p38alpha kinase | 10 | 8 |  |  |
| PDE2A | 10 | 9 |  |  |
| PDE3B | 10 | 4 |  |  |
| PDE4D | 10 | 11 |  |  |
| PDE4 rolipram binding | 10 | 10 |  |  |
| PDE5 | 10 | 7 |  |  |
| PDE6 | 10 | 7 |  |  |
| PDE11 | 10 | 6 |  |  |
| PPAR gamma | 10 | 60 | 2.8 | no agonist or antagonist activity at 10 μM |
| Serotonin 5-HT transporter | 10 | 0 |  |  |
| Serotonin 5-HT1A | 10 | 13 |  |  |
| Serotonin 5-HT1B | 10 | 6 |  |  |
| Serotonin 5-HT2A | 10 | -2 |  |  |
| Serotinin 5-HT2B | 10 | -6 |  |  |
| Serotonin 5-HT2C | 10 | -4 |  |  |
| Serotonin 5-HT2C | 10 | 4 |  |  |
| Serotonin 5-HT3 | 10 | 4 |  |  |
| Serotonin 5-HT4 | 10 | -7 |  |  |
| Serotonin 5-HT6 | 10 | 2 |  |  |
| Serotonin 5-HT7 | 10 | -7 |  |  |
| Sigma receptor | 10 | -8 |  |  |
| Somatostatin receptor 4 | 10 | 5 |  |  |
| TRPV1 | 20 |  |  | No agonist or antagonist activity at 20 μM |
| Urotensin receptor | 10 | 32 |  |  |
